# The developing epicardium regulates cardiac chamber morphogenesis by promoting cardiomyocyte growth

**DOI:** 10.1242/dmm.049571

**Published:** 2022-10-19

**Authors:** Giulia L. M. Boezio, Shengnan Zhao, Josephine Gollin, Rashmi Priya, Shivani Mansingh, Stefan Guenther, Nana Fukuda, Felix Gunawan, Didier Y. R. Stainier

**Affiliations:** ^1^ Department of Developmental Genetics, Max Planck Institute for Heart and Lung Research, 61231 Bad Nauheim, Germany; ^2^ DZHK German Centre for Cardiovascular Research, Partner Site Rhine-Main, 61231 Bad Nauheim, Germany; ^3^ Cardio-Pulmonary Institute, Aulweg 130, 35392 Giessen, Germany; ^4^ Bioinformatics and Deep Sequencing Platform, Max Planck Institute for Heart and Lung Research, 61231 Bad Nauheim, Germany

**Keywords:** Cardiomyocytes, Cell growth, Epicardium, Heart development, Inter-tissue crosstalk, Zebrafish

## Abstract

The epicardium, the outermost layer of the heart, is an important regulator of cardiac regeneration. However, a detailed understanding of the crosstalk between the epicardium and myocardium during development requires further investigation. Here, we generated three models of epicardial impairment in zebrafish by mutating the transcription factor genes *tcf21* and *wt1a*, and ablating *tcf21^+^* epicardial cells. Notably, all three epicardial impairment models exhibited smaller ventricles. We identified the initial cause of this phenotype as defective cardiomyocyte growth, resulting in reduced cell surface and volume. This failure of cardiomyocyte growth was followed by decreased proliferation and increased abluminal extrusion. By temporally manipulating its ablation, we show that the epicardium is required to support cardiomyocyte growth mainly during early cardiac morphogenesis. By transcriptomic profiling of sorted epicardial cells, we identified reduced expression of FGF and VEGF ligand genes in *tcf21^−/−^* hearts, and pharmacological inhibition of these signaling pathways in wild type partially recapitulated the ventricular growth defects. Taken together, these data reveal distinct roles of the epicardium during cardiac morphogenesis and signaling pathways underlying epicardial-myocardial crosstalk.

## Teaser

**Summary:** The epicardium is required to promote cardiomyocyte growth during ventricular development at least in part via the FGF and VEGF signaling pathways.

## INTRODUCTION

The epicardium is the last layer to incorporate into the heart during development. Epicardial cells (EpiCs) delaminate from the extra-cardiac proepicardial organ (PEO) and attach to the naked myocardium as free-floating cells owing to the physical properties of the pericardial fluid (Rodgers et al., 2008; Peralta et al., 2013). The epicardium forms a mesothelial layer that completely envelops the heart, then undergoes epithelial-to-mesenchymal transition (EMT) and gives rise to various epicardial derived cells (EPDCs) (Smith et al., 2011; Acharya et al., 2012; Smits et al., 2018). Although the epicardium becomes dormant after undergoing EMT, it reactivates after cardiac injury and upregulates developmental genes as well as new gene regulatory networks (Weinberger et al., 2021 preprint), and subsequently regenerates (van Wijk et al., 2012; Masters and Riley, 2014; Cao and Poss, 2018).

The epicardium has received great attention because of its ability to differentiate into a multitude of cell types during cardiac repair and its role as a source of paracrine signals that promote wound healing (Masters and Riley, 2014; Cao and Poss, 2018). However, a detailed understanding of the epicardial-myocardial crosstalk has proven more elusive. As the factors involved in epicardial-myocardial signaling identified to date, including components of the fibroblast growth factor (FGF) and insulin growth factor (IGF) signaling pathways, are expressed in both developmental and regenerative contexts (Pennisi et al., 2003; Lavine et al., 2005; Brade et al., 2011; Li et al., 2011; Vega-Hernandez et al., 2011), identifying the processes underlying epicardial-myocardial crosstalk during development has important implications for cardiac repair.

Defects in epicardial coverage consistently result in a common myocardial phenotype – small, underdeveloped ventricles. Ablation of the PEO in chicken embryos causes reduced cardiac size and occasional ventricular bulging (Manner, 1993; Pennisi et al., 2003; Manner et al., 2005; Takahashi et al., 2014). Similarly, in mouse embryos, mutations in several epicardial-enriched genes, including those encoding the transcription factors TCF21 and WT1, abrogate epicardial coverage, leading to a reduction in ventricular size (Moore et al., 1999; Acharya et al., 2012). Most studies to date have concluded that the major role of the epicardium is to promote cardiomyocyte (CM) proliferation (Pennisi et al., 2003; Lavine et al., 2005; Li et al., 2011) and to contribute to the ventricular mass by giving rise to EPDCs such as fibroblasts (Mahtab et al., 2009; Acharya et al., 2012). Notably, a few studies have started to challenge the view that the sole function of the epicardium is to regulate the CM cell cycle (Eid et al., 1992; Kastner et al., 1994; Takahashi et al., 2014), but they have so far been limited to using *in vitro* explants or fixed tissue sections. Deeper investigation of epicardial function in promoting myocardial growth requires a model in which the cellular phenotypes can be experimentally followed in four dimensions.

Here, we generated three models of epicardial impairment in zebrafish larvae by mutating the transcription factor genes *tcf21* and *wt1a*, and by ablating EpiCs. Leveraging the advantages of these newly established models and the amenability of zebrafish to live imaging at a three-dimensional (3D) resolution, we identified a novel role for the epicardium in promoting CM growth and determined the time window when this epicardium-to-myocardium interaction occurs. We also generated a transcriptomic dataset of epicardial-enriched factors to identify molecules important for this crosstalk. Focusing on *fgf24* and *vegfaa*, we provide evidence that they are epicardial-enriched regulators of ventricular growth.

## RESULTS AND DISCUSSION

### Generation of three zebrafish epicardial impairment models

**Fig. 1.**
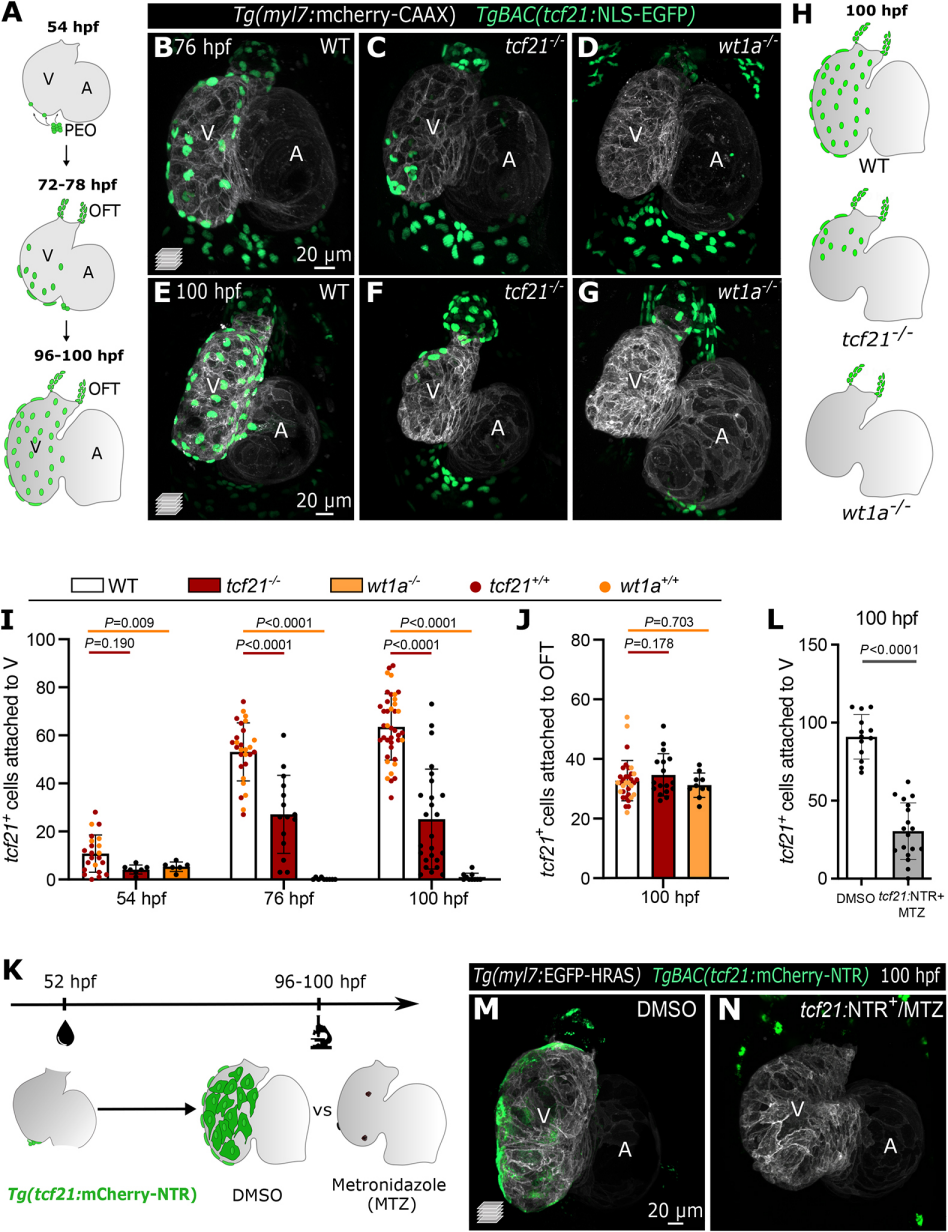
**The transcription factors Tcf21 and Wt1a are required for epicardial attachment to the ventricle.** (A) Schematic representation of the epicardial coverage of the zebrafish embryonic and larval heart. (B-G) Confocal images of 76 hpf (B-D) and 100 hpf (E-G) *Tg(myl7:mCherry-CAAX) TgBAC(tcf21:NLS-EGFP)* larvae. (H) Schematics of the epicardial coverage in 100 hpf WT, *tcf21^−/−^* and *wt1a^−/−^* larvae. Gray, myocardium; green, EpiCs. (I-J) Quantification of *tcf21^+^* EpiCs attached to the ventricular myocardium (I) and OFT (J). The colors of WT dots refer to *tcf21^+/+^* (red) and *wt1a^+/+^* (orange) siblings. Data show the mean±s.d. *P-*values were determined from unpaired two-tailed *t-*test or Mann–Whitney test (following normality test) compared with +/+ siblings of each genotype. (K) Epicardial ablation protocol using the NTR/MTZ system. (L) Quantification of *tcf21^+^* EpiCs attached to the ventricular myocardium following epicardial ablation. Data show the mean±s.d.; *P-*values were determined from unpaired two-tailed *t-*test. Controls are pooled *tcf21:*NTR^+^ DMSO-treated and *tcf21:*NTR^−^ MTZ-treated larvae (see Materials and Methods). (M,N) Confocal images of 100 hpf *Tg(myl7:EGFP-HRAS); TgBAC(tcf21:mCherry-NTR)* hearts showing the absence of EpiCs post MTZ treatment (N), compared with DMSO-treated larvae (M). WT, wild type; A, atrium; V, ventricle; OFT, outflow tract; PEO, proepicardial organ.

In zebrafish, EpiCs start adhering to the myocardial wall around 52-56 h post fertilization (hpf), and cover the entire ventricle by 96-120 hpf (Fig. 1A) (Peralta et al., 2014). To study the epicardial-myocardial crosstalk, we generated three different zebrafish models with impaired epicardial coverage. First, we mutated *tcf21* and *wt1a* (Fig. S1C), two transcription factor genes enriched in the epicardium (Fig. S1A,B) (Serluca, 2008; Liu and Stainier, 2010; Peralta et al., 2014)). Our *tcf21* deletion allele contains a frameshift in the coding sequence, leading to a predicted truncated protein with an incomplete DNA-binding domain (Fig. S1C), whereas the mutation did not affect the stability of the mutant mRNA (Fig. S1D). Conversely, our *wt1a* promoter deletion led to a complete absence of *wt1a* mRNA (Fig. S1C,E). In mouse, the lack of either transcription factor leads to impaired epicardial coverage, but its impact on myocardial development is poorly understood (Moore et al., 1999; Acharya et al., 2012). We observed that in zebrafish, mutations in *tcf21* and *wt1a* also led to a reduction of *TgBAC(tcf21:*NLS-EGFP*)^+^* (hereafter referred to as *tcf21^+^*) EpiCs on the ventricular wall, as evidenced at 54 hpf and even more prominently at 76 and 100 hpf (Fig. 1B-H). However, whereas *wt1a* mutants exhibited a complete absence of *tcf21^+^* EpiCs on the ventricular wall, the epicardial coverage reduction in *tcf21* mutants was variable (Fig. 1I). This phenotypic variability is likely not due to the *tcf21* mutation leading to a hypomorphic allele, as non-cardiac phenotypes previously identified in *tcf21* mutants, including the lack of head muscles (Nagelberg et al., 2015; Burg et al., 2016), were observed with complete penetrance in our *tcf21* mutants (*n*>300 larvae). Notably, the number of outflow tract (OFT) *tcf21^+^* EpiCs appeared to be unaffected in both mutants (Fig. 1J), likely owing to the different origin of this epicardial population (Perez-Pomares et al., 2003; Weinberger et al., 2020). Next, to establish a model in which epicardial coverage could be impaired in a specific time window, we used the previously described nitroreductase/metronidazole (NTR/MTZ) system (Curado et al., 2007, 2008; Pisharath et al., 2007). By treating *TgBAC(tcf21:mCherry-NTR)* (Wang et al., 2015) embryos (*tcf21:*NTR^+^) with MTZ before the epicardium covered the ventricle (52-100 hpf; Fig. 1K), we could ablate a majority of the *tcf21^+^* EpiCs (∼67%; Fig. 1L-N), thereby establishing an inducible system that complements our two mutant models. Using the pan-epicardial marker caveolin 1 (Cav1) (Cao et al., 2016), we confirmed that *TgBAC(tcf21:*NLS-EGFP*)* expression was a reliable marker for all EpiCs, and that the loss of *tcf21^+^* cells in our models was due to an absence of EpiCs and not the loss of *TgBAC(tcf21:*NLS-EGFP*)* expression. Cav1 immunostaining was only present in ‘escaper’ ventricular *tcf21^+^* EpiCs in *tcf21^−/−^* hearts, and around the OFT in all three models (Fig. S1F-I′). Altogether, these three distinct epicardial impairment models constitute a complementary set of reagents to study the effects of epicardial impairment on cardiac morphogenesis. Moreover, the genetic models generated here will be useful to further investigate how Wt1a and Tcf21 regulate epicardial development before the onset of EMT.

### Impairment in cardiomyocyte growth becomes evident before reduced cardiomyocyte proliferation when epicardial cells are lost

**Fig. 2.**
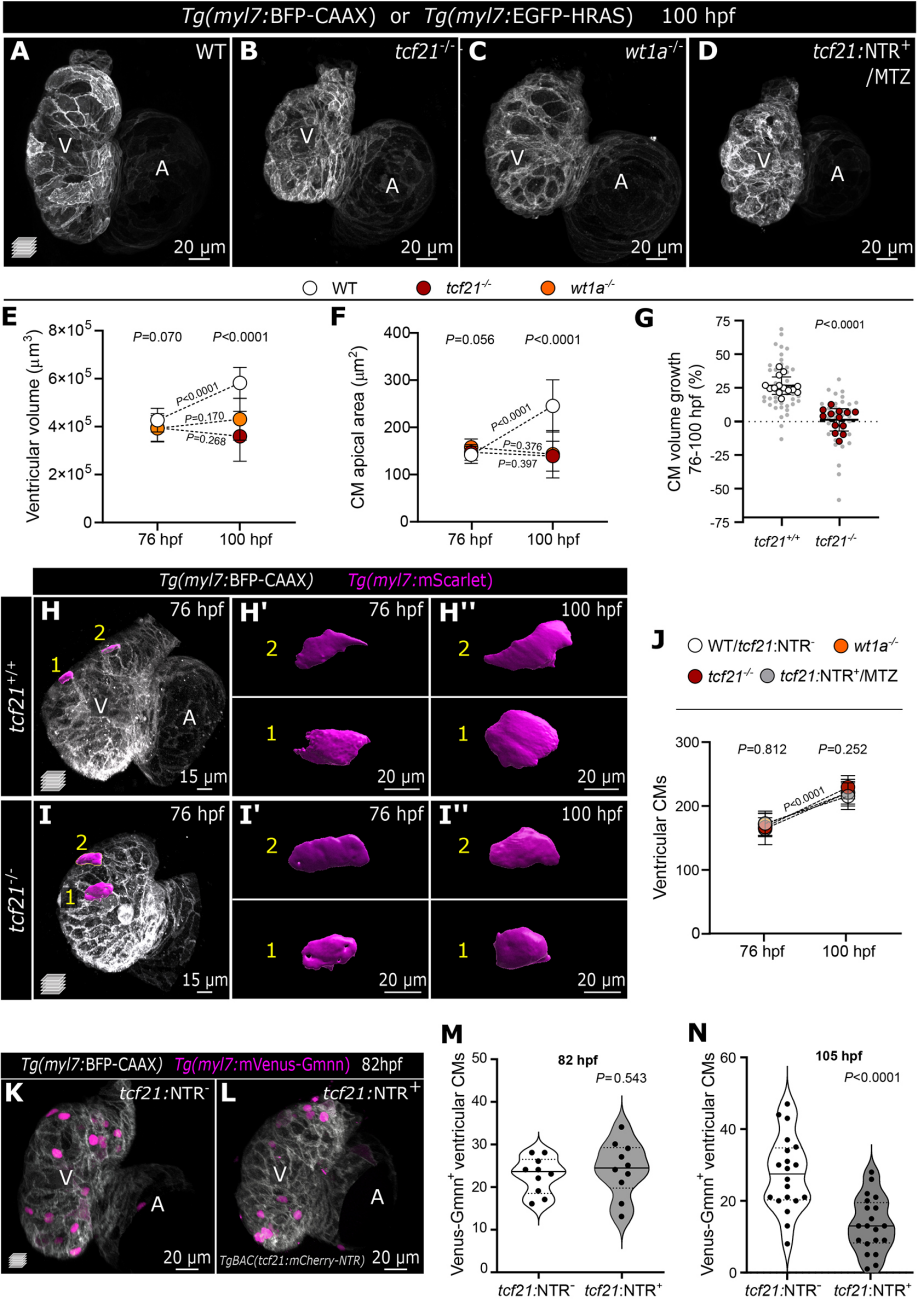
**Impaired epicardial coverage affects ventricular cardiomyocyte size increase and ventricular growth.** (A-D) Confocal images of 100 hpf WT, *tcf21^−/−^*, *wt1a^−/−^* and *TgBAC(tcf21:mCherry-NTR)^+^* (*tcf21:*NTR^+^) MTZ-treated larvae, exhibiting reduced ventricular size. (E,F) Change in ventricular volume (E) and CM apical area (F) from 76 to 100 hpf in WT, *tcf21^−/−^* and *wt1a^−/−^* larvae. (G) Percentage increase of individual CM volume between 76 and 100 hpf, measured by the *myl7:*mScarlet signal. Large dots indicate the average per larva and small dots indicate individual CMs. *P-*values were determined from unpaired two-tailed *t-*test comparing the averages per larva. (H-I″) Confocal images of *Tg(myl7:BFP-CAAX)*; *tcf21^+/+^* or *tcf21^−/−^* larva at 76 and 100 hpf (same larvae shown), injected with *myl7:mScarlet* DNA to label individual CMs. (H′-I″) 3D surface rendering of individual CMs at the two time points. (J) Changes in ventricular CM numbers from 76 to 100 hpf in WT (or *tcf21:*NTR^−^), *tcf21^−/−^*, *wt1a^−/−^* and *tcf21:*NTR^+^-MTZ treated larvae. (K-N) Confocal images and quantification of *myl7:*mVenus-Gmnn*^+^* CMs in 82 hpf (K-M) and 105 hpf (N) control (*tcf21:*NTR^−^) and *tcf21:*NTR^+^ MTZ-treated larvae. *P*-values (M,N) were determined by unpaired two-tailed *t-*test. Data show the mean±s.d. (E,F,G,J), and the solid and dotted lines (M,N) indicate the median and quartiles, respectively. *P-*values from one-way ANOVA among the three different genotypes at the same time point are shown above the graph (E,F,J), or from unpaired two-tailed *t-*test comparing the two different time points within the same genotype are shown on the dotted lines (E,F,J). Single data points are shown in Fig. S2. WT, wild type; A, atrium; V, ventricle.

We next aimed to determine how epicardial impairment affects ventricular morphogenesis. Starting at 96 hpf, we observed pericardial edema in *tcf21^−/−^*, *wt1a^−/−^* and *tcf21:*NTR^+^ MTZ-treated larvae (Fig. S1J-M), together with impaired ventricular fractional shortening starting at 100 hpf (Fig. S2D). The cardiac ventricle in all epicardial-impairment models was also approximately 30% smaller than in wild type (WT; Fig. 2A-D; Fig. S2A-E). Interestingly, we observed that, from 76 to 100 hpf, the WT ventricle grew on average by 36.4% in volume, whereas the mutant ventricle started with a comparable volume at 76 hpf but failed to enlarge over time (Fig. 2E).

Increase in organ size is driven by hypertrophic (increase in cell size) and hyperplastic (increase in cell number) growth. The first phenotype we observed between control and epicardial-impairment models was in the CM apical area. Although the average apical area of compact-layer CMs was comparable in WT and mutant larvae at 76 hpf, it was significantly smaller in mutant CMs compared with WT at 100 hpf (Fig. 2F; Fig. S2F). To assess the volumetric growth of individual compact-layer CMs over time, we tracked single CMs in *tcf21^+/+^* and *tcf21^−/−^* hearts by mosaic expression of *Tg(myl7:mScarlet)*. Strikingly, *tcf21^+/+^* CM volume increased by 26.8% between 76 and 100 hpf, whereas *tcf21^−/−^* CM volume did not significantly change (+1.4%) (Fig. 2G-I). Our data, possibly for the first time, clearly reveal a correlation between increasing CM cell volume and ventricular growth and uncover a requirement for the epicardium in promoting CM cell growth during development.

Although the epicardium has not been previously linked with CM hypertrophic growth, it has been implicated in promoting CM proliferation (Pennisi et al., 2003; Lavine et al., 2005; Li et al., 2011). We therefore assessed the number of proliferating CMs at 82 hpf, a time point before the growth defects can be observed, by counting the number of Venus-Gmnn^+^ CMs (i.e. CMs in the S/G2/M phases; Sugiyama et al., 2009; Choi et al., 2013). We observed no significant difference between control and *tcf21:*NTR^+^ MTZ-treated larvae (Fig. 2K-M). We also counted the number of ventricular CMs and they increased by a similar proportion (∼30%) in WT larvae and in larvae from all three models between 76 and 100 hpf (Fig. 2J), resulting in comparable numbers of CMs at all time points analyzed (Fig. S2G). In addition, the loss of the epicardium did not affect the number of CMs in the trabecular layer as assessed in 100 hpf *tcf21^−/−^* hearts (*P=*0.307, unpaired two-tailed *t*-test; Fig. S2H). These data support the hypothesis that the impaired ventricular growth observed in the absence of the epicardial layer is not caused by defects in CM numbers or differences in their trabeculation potential. However, at 105 hpf, a time point subsequent to the appearance of CM size defects, we observed a severe reduction in the number of Venus-Gmnn*^+^* CMs in *tcf21:*NTR^+^ MTZ-treated larvae compared with controls (∼48.5%; Fig. 2N). These defects in CM proliferation might be a consequence of the initial impairment in CM growth. In support of this interpretation, eukaryotic cell cycle progression is known to depend on cell growth (Jorgensen and Tyers, 2004), and a multitude of cell types, including CMs, expand in size prior to dividing (Son et al., 2015; Zlotek-Zlotkiewicz et al., 2015; Uribe et al., 2018). In addition, we observed CM extrusion away from the cardiac lumen in the three epicardial impairment models (Fig. S3A-F′), consistent with previous reports (Manner et al., 2005; Rasouli et al., 2018). The extrusion of these CMs does not appear to be caused by increased cell death, as evidenced by their intact nuclei (Fig. S3G-G‴), nor from a change in their fate to epicardial cells (Fig. S4E, *n*>30 at 76 and 100 hpf), as recently reported in a model of *wt1* expressing CMs (Marques et al., 2022). CM extrusion has also been observed in zebrafish *snai1b* mutants, in which extruding CMs eventually detach and are found in the pericardial cavity (Gentile et al., 2021); whether epicardial cells also play a role in this model remains to be determined. Notably, we observed a reduced internuclear distance between CMs in our epicardial impairment models (Fig. S3H-J), which has not been reported in other models of CM extrusion. Therefore, we postulate that in the absence of the epicardium, excessive cellular density in the ventricular myocardium drives the aberrant extrusion of a few (i.e. fewer than ten) CMs. Altogether, our observations uncover a previously unidentified role for the epicardium in promoting the initial stages of CM growth, which affects ventricular growth as well as CM proliferation.

### Epicardial cells are required and sufficient for ventricular cardiomyocyte growth in a restricted early time window

**Fig. 3.**
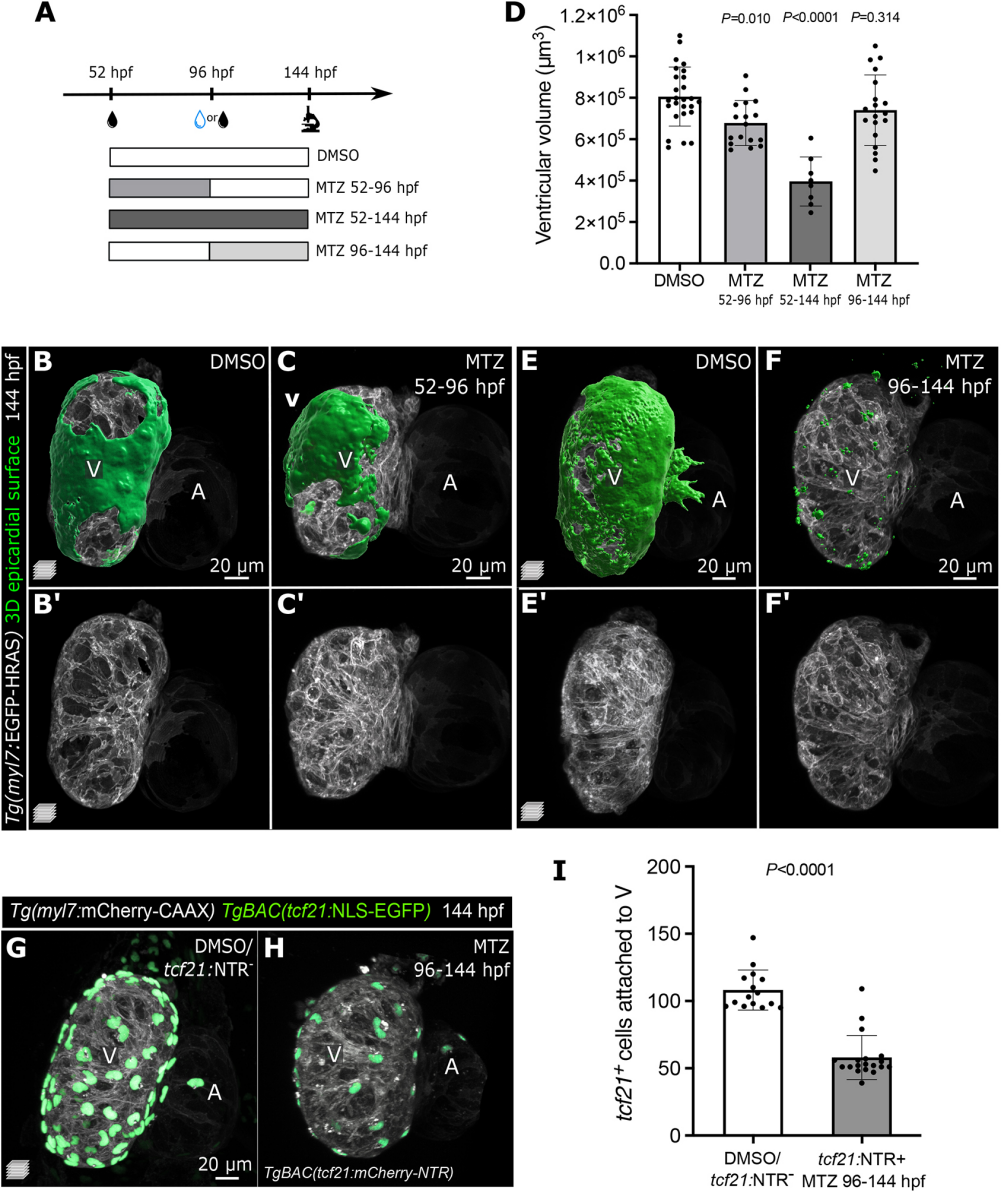
**Epicardial cells are required for ventricular cardiomyocyte growth during early cardiac morphogenesis, but are dispensable at later time points.** (A) Schematic of the MTZ treatment protocol. (B-C′,E-F′) Confocal images of 144 hpf *Tg(myl7: EGFP-HRAS); TgBAC(tcf21:mCherry-NTR)* DMSO and MTZ-treated larvae. Green, 3D reconstruction of epicardial coverage showing a partial recovery of EpiCs in 52-96 hpf MTZ-treated larvae, and a clear reduction in 96-144 hpf MTZ-treated larvae. (D) Quantification of ventricular volume at 144 hpf following epicardial regeneration or late epicardial ablation. (G-I) Confocal images and quantification of 144 hpf *Tg(myl7:mCherry-CAAX); TgBAC(tcf21:NLS-EGFP); TgBAC(tcf21:mCherry-NTR)* DMSO and MTZ-treated larvae, showing the reduction of *tcf21^+^* EpiCs attached to the ventricular myocardium after their late-onset ablation. Controls were pooled, including *tcf21:*NTR^+^ DMSO-treated and *tcf21:*NTR^−^ MTZ-treated larvae (see Materials and Methods). Data show the mean±s.d. (D,I) and *P-*values are from one-way ANOVA with Dunnett's post hoc test (D) or unpaired two-tailed *t-*test (I). A, atrium; V, ventricle.

To further analyze the dependency of CM growth on the epicardium, we tested whether rescuing the epicardial coverage was sufficient to improve ventricular growth. Leveraging the temporal versatility of the NTR/MTZ system, we ablated the epicardium specifically from 52 to 96 hpf and then washed out the MTZ. We first confirmed that the EpiCs recovered by 144 hpf (Fig. 3A-C), as previously reported (Wang et al., 2015). Notably, epicardial restoration was sufficient to ameliorate the cardiac growth defects in MTZ-treated larvae (Fig. 3B′-D).

Previous studies investigating the consequences of reduced epicardial coverage have used genetic models or physical ablation of the PEO, but the constitutive lack of the epicardium fails to pinpoint the time window in which EpiCs promote myocardial development. As mentioned above, we identified the 52-96 hpf window to be crucial for epicardial-myocardial interactions, which coincides with the period of epicardial attachment. We then tested the effects of epicardial ablation between 96 and 144 hpf (Fig. 3A). Surprisingly, although the reduction in epicardial coverage was significant (Fig. 3E-I), we did not observe any obvious morphological defects in ventricular morphology or size (Fig. 3D-F′).

Taken together, these results suggest that an epicardial-myocardial crosstalk is necessary to regulate ventricular volume at the early epicardial attachment stage (52-96 hpf), but dispensable once epicardial coverage is complete (>96 hpf). We propose that from 96 hpf onwards, CMs continue to grow due to epicardial-independent intrinsic or extrinsic cues. Therefore, at later stages, the epicardium might assume a different function, including preparing for the onset of EMT. Future investigations of later epicardial function during cardiac development (e.g. at the onset of EMT and EPDC formation) will greatly benefit from the temporal versatility of this *tcf21:*NTR/MTZ model.

### Epicardial-derived secreted factors promote ventricular cardiomyocyte growth

Next, we aimed to understand how the epicardium promotes CM growth. The epicardium is an important signaling center during development and regeneration (Quijada et al., 2020). Nonetheless, the appearance of extruding CMs in epicardial-deficient larvae (Fig. S3), which was also observed following PEO ablation in chick (Manner et al., 2005), raised questions as to whether the epicardium primarily acts as a physical barrier to preserve myocardial integrity. To further investigate the role of the epicardium as a mechanical support and/or signaling source, we focused on the sizable subgroup of *tcf21^−/−^* hearts with substantial epicardial coverage on their ventricle (Fig. 1H; Fig. S4A-C). We first observed that these mutants display defects in ventricular size comparable with those in *tcf21^−/−^* hearts devoid of epicardial coverage (Fig. S4A-C). We found no significant correlation between the number of ventricular EpiCs and ventricular volume (Fig. S4D), or the number of extruding CMs (Fig. S4F). Notably, we also observed that, occasionally, extruding CMs were covered by EpiCs (Fig. S4E,E′,G-G″), suggesting that the physical presence of EpiCs alone does not prevent CM extrusion.

**Fig. 4.**
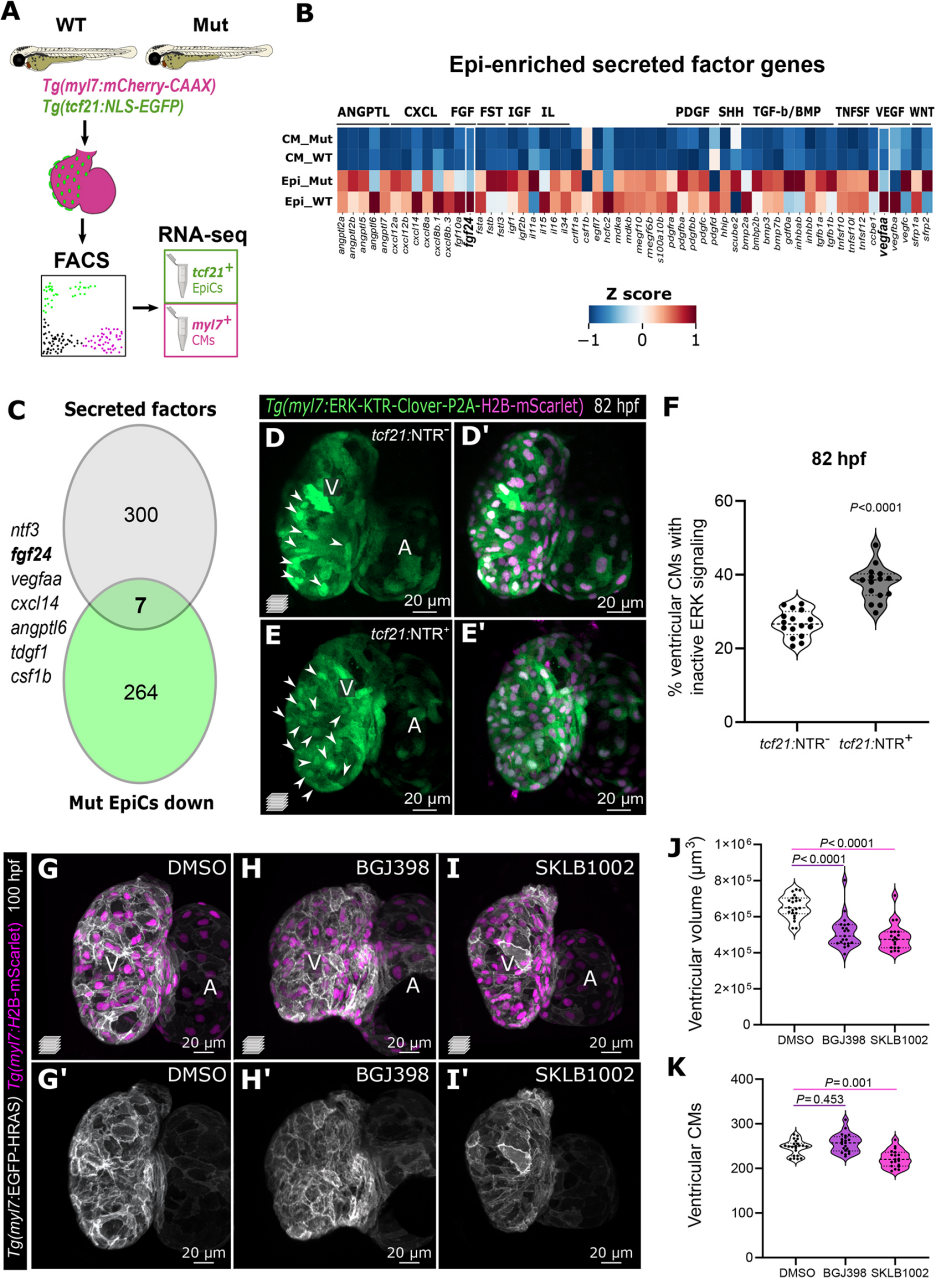
**Epicardial-enriched FGF and VEGF ligand genes in epicardial-myocardial crosstalk.** (A) RNA-seq from *tcf21^+^* and *myl7^+^* cells from 96 hpf *tcf21^+/+^* and *tcf21^−/−^* hearts. (B) Heatmap of zebrafish secreted factor genes in *tcf21^+/+^* and *tcf21^−/−^* EpiCs and CMs, showing Z-scores normalized per row. The genes highlighted in bold are differentially expressed between *tcf21^+/+^* and *tcf21^−/−^* EpiCs (log_2_FC<−0.7 or >0.7, *P*_adj_<0.05). (C) Venn diagram denoting the intersection between secreted factor genes in the zebrafish genome and genes that are downregulated (log_2_FC<−0.7, *P*_adj_<0.05) in *tcf21^−/−^* EpiCs. (D-F) Confocal images and quantification of 82 hpf *Tg(myl7:ERK-KTR-Clover-P2A-H2B-mScarlet)* ventricles in control (*tcf21:*NTR^−^) and *TgBAC(tcf21:mCherry-NTR)* (*tcf21:*NTR^+^) larvae treated with MTZ. White arrowheads point to CMs with nuclear Clover (inactive ERK), quantified in F. (G-K) Confocal images (G-I′), quantification of ventricular volume (J) and CM numbers (K) of 100 hpf larvae treated with FGFR (BGJ398) and VEGFR (SKLB1002) inhibitors starting at 65 hpf. The solid and dotted lines (F,J,K) indicate the median and quartiles, respectively, and *P*-values are from Kruskal-Wallis test with Dunn's post-hoc test. A, atrium; V, ventricle.

To identify epicardial factors necessary for CM growth, we compared the transcriptomes of sorted *tcf21^+/+^* and *tcf21^−/−^* EpiCs and CMs at 96 hpf (Fig. 4A; Fig. S5A-C; Tables S2 and S3). To minimize bias in the analyses, we selected *tcf21^−/−^* larvae with similarly substantial ventricular epicardial coverage and collected the same number of EpiCs from the two genotypes (see Materials and Methods). We first determined the genes expressed in the two WT populations by RNA-sequencing (RNA-seq) and confirmed the expression of known cell type-specific markers, including *postnb*, *wt1a*, *col1a2*, *cav1* and *aldh1a2* in EpiCs and *ttn.1*, *ttn.2*, *myh7* and *myh6* in CMs. We also observed that the most enriched Gene Ontology (GO) terms reflected the biological processes prominent in the two cell types (e.g. EpiCs – extracellular matrix secretion, cell adhesion; CMs – mitochondrial processes and metabolism; Fig. S5C). Comparing the WT and *tcf21^−/−^* populations, we observed that genes upregulated in *tcf21^−/−^* EpiCs encode multiple extracellular matrix (ECM) components (including various collagens, as well as Postna and Has) and the related GO terms were amongst the most enriched (Fig. S5E). We envision that this dataset will be an important resource to further investigate epicardial development and regulation of epicardial function during embryogenesis.

To identify potential molecular mechanisms disrupted in *tcf21^−/−^* hearts leading to the lack of CM growth, we explored GO terms that were enriched in the downregulated genes in *tcf21^−/−^* CMs (Fig. S5F). From this analysis, mitochondrial and ribosome-related terms were the most over-represented groups (Fig. S5F,G). In particular, genes that encode several mitochondrial ribosomal proteins (MRPs), which are necessary for mitochondrial protein synthesis and mitochondrial activity (Sylvester et al., 2004), were downregulated in *tcf21^−/−^* CMs. These data suggest that the defect in CM volume increase may be due to the failure of mitochondria to maintain normal metabolic function. Mutations in some MRP genes are correlated with cardiomyopathies in the human population (Huang et al., 2020; Friederich et al., 2021); however, further analysis is necessary to investigate how defects in MRP function lead to cardiac phenotypes.

For the purpose of this study, we decided to focus on epicardial-derived secreted factors that potentially mediate epicardial-myocardial crosstalk. Among the factors enriched in the EpiCs compared with CMs are members of the FGF, IGF, transforming growth factor (TGF)-β, and platelet-derived growth factor (PDGF) pathways (Fig. 4B; Table S3). These pathways are important for epicardial-myocardial crosstalk in mice (Olivey and Svensson, 2010; Li et al., 2017), suggesting that the molecular regulators of this crosstalk might be similar in zebrafish and humans.

Among the downregulated secreted factors in *tcf21^−/−^* EpiCs are FGF and VEGF ligand genes, including *fgf10a*, *fgf24*, *vegfaa* and *vegfba* (Fig. 4B,C; Table S3)*. fgf24* and *vegfaa* (Fig. 4C; Fig. S6A), in particular, are the FGF and VEGF ligand genes with the highest epicardial expression in our dataset and the only ones significantly downregulated (*P*_adj_<0.05; Table S3)*.* The FGF pathway mediates epicardial-myocardial crosstalk in mouse and chicken embryos, where it is primarily known for its role in promoting CM proliferation (Pennisi et al., 2003; Lavine et al., 2005). By *in situ* hybridization, we observed *fgf24* expression in 76 hpf hearts (Fig. S6B,B′), and it appeared to be enriched in the epicardium (Fig. S6C-C‴). Vegfaa promotes angiogenesis and coronary vessel formation (Liang et al., 2001; Wu et al., 2012; Marin-Juez et al., 2016; Rossi et al., 2016), but its role in the developing epicardium has not been investigated until recently (Bruton et al., 2022). We observed that *vegfaa* expression in the developing heart appeared to be initially limited to the epicardium until 100 hpf (Fig. S6D-E″; Bruton et al., 2022), at which point it was also expressed in the endocardium (Karra et al., 2018). The epicardial enrichment of *fgf24* and *vegfaa* expression {log_2_[fold change (FC)]: +4.77 and +3.38, respectively} as well as their downregulation in *tcf21^−/−^* EpiCs led us to hypothesize that epicardial-derived Fgf24 and Vegfaa mediate signaling to the myocardium. To test this hypothesis, we first assessed the activation of the mitogen-activated protein kinase (MAPK)/extracellular regulated kinase (ERK) pathway, a downstream effector of both FGF and VEGF signaling, in CMs. We used the recently generated transgenic line *Tg(myl7:ERK-KTR-Clover-P2A-H2B-mScarlet)* (Qi et al., 2022) to monitor ERK activation through a kinase translocation reporter (de la Cova et al., 2017; Mayr et al., 2018; Okuda et al., 2021) (Fig. 4D). Following MTZ treatment between 52 and 80 hpf, *Tg(myl7:ERK-KTR-Clover-P2A-H2B-mScarlet); TgBAC(tcf21:mCherry-NTR)* hearts devoid of the epicardium exhibited a significantly increased number of CMs with inactive ERK signaling compared with control hearts (Fig. 4D-F). In addition, we used BGJ398 (De Simone et al., 2021) and SKLB1002 (Zhang et al., 2011) to broadly inhibit the FGF and VEGF signaling pathways, respectively, in a defined time window (65 to 100 hpf). We chose 65 hpf to avoid phenotypes caused by inhibiting the earlier roles of FGF and VEGF (Haigh, 2008; Khosravi et al., 2021). Notably, larvae treated with these compounds recapitulated the small ventricle phenotype observed in the epicardial-deficient hearts. FGF inhibition affected ventricular volume without causing any changes in CM numbers, whereas the VEGF inhibitor led to a mild decrease in ventricular CMs (∼10%; Fig. 4G-K). Thus, global inhibition of the VEGF pathway by SKLB1002 appeared to cause a stronger cardiac phenotype compared with the epicardial-specific downregulation of *vegfaa* in *tcf21^−/−^* hearts, and possibly affected additional signaling pathways.

Interestingly, the mammalian ortholog of Fgf24 binds Fgfr4 (Mok et al., 2014), which is highly expressed in zebrafish CMs and downregulated in *tcf21^−/−^* CMs, as per our transcriptomic datasets (Table S2). We speculate that the downregulation of Fgfr4 in *tcf21^−/−^* CMs might in part be due to the reduction in Fgf24 ligand in these mutants, and additional studies will be needed to investigate the involvement of different FGF ligands and receptors in regulating CM growth. However, Vegfaa is not known to bind receptors prominently expressed in CMs but binds Vegfr2, which is enriched in EpiCs. Therefore, Vegfaa might signal to EpiCs in an autocrine manner, similar to retinoic acid (Stuckmann et al., 2003; Brade et al., 2011), and regulate the production of other signaling molecules. It was also recently proposed that the epicardial expression of *vegfaa* (in response to macrophage activation) regulates Notch activity in the endocardium, which in turn signals to CMs (Bruton et al., 2022). A few studies have linked human FGF21 to mitochondrial function and disease (Tezze et al., 2019), but a clear mechanism is lacking.

Determining and comparing the molecular pathways impacted in each of the three different epicardial impairment models we present, through transcriptomic analysis for example, should give important insights into the epicardial-regulated mechanisms underlying CM growth. Also, we cannot exclude the possibility that the secreted factors that are upregulated in *tcf21^−/−^* hearts, such as *il1a*, *scube2*, *ccbe1* and *sfrp2*, are involved in driving the cardiac phenotype. Further studies are needed to address the molecular mechanisms by which these signaling pathways mediate epicardial-myocardial crosstalk and how, in turn, this interaction promotes ventricular morphogenesis.

It is also likely that ECM-related components secreted by the epicardium play a role in CM growth and homeostasis. In particular, our transcriptomic analysis points to an altered ECM composition with the downregulation of some ECM genes, including *col6a1*, *col6a2*, *col4a1*, *col4a2*, *lama5*, *hapln1a* and *hapln1b*, and the upregulation of several other collagen isoforms, as well as *postna*, *has* and several metalloproteinases*.* Thus, it will also be interesting to investigate the potential role of the epicardial-derived ECM in promoting myocardial growth.

Taken together, our data uncover a previously unappreciated requirement for the epicardium in promoting CM growth at the cellular and tissue levels, which takes place prior to its previously reported role in stimulating CM proliferation. Moreover, we provide evidence that this inter-tissue crosstalk is mediated by the FGF and VEGF signaling pathways. The three epicardial-impairment models generated for and used in this study provide genetically tractable and, in one case, temporally manipulable systems that complement existing models to deepen our understanding of the cellular and molecular processes involved in epicardial-myocardial crosstalk during cardiac development.

## MATERIALS AND METHODS

### Zebrafish husbandry and lines

All zebrafish husbandry was performed under standard conditions, and all experiments were conducted in accordance with institutional (Max-Planck Gesellschaft) and national ethical and animal welfare guidelines. The following lines were used in the study: *Tg(-0.8myl7:nlsDsRedExpress)hsc4*, abbreviated *Tg(myl7:nlsDsRed)* (Takeuchi et al., 2011); *Tg(myl7:mVenus-gmnn)ncv43* (Jimenez-Amilburu et al., 2016); *Tg(myl7:EGFP-Hsa.HRAS)s883*, abbreviated *Tg(myl7:EGFP-HRAS)* (D'Amico et al., 2007); *TgBAC(tcf21:mCherry-NTR)pd108* (Wang et al., 2015); *TgBAC(tcf21:NLS-EGFP)pd41* (Wang et al., 2015); *Tg(myl7:BFP-CAAX)bns193* (Guerra et al., 2018); *TgBAC(vegfaa:EGFP)pd260* (Karra et al., 2018); *Tg(myl7:mCherry-CAAX)bns7* (Uribe et al., 2018); *Tg(-0.8myl7:ERK-KTR-Clover-P2A-H2B-mScarlet)bns565*, abbreviated *Tg(myl7:ERK-KTR-Clover-P2A-H2B-mScarlet)* (Qi et al., 2022); *Tg(-0.8myl7:H2B-mScarlet)bns534*, abbreviated *Tg(myl7:H2B-mScarlet)* (this study); *tcf21^bns427^* (this study); and *wt1a^bns428^* (this study). All eggs, embryos and larvae were kept in egg water (3 g Instant Ocean, 0.75 g calcium sulfate, 10 l distilled water) unless otherwise stated.

### Generation of transgenic lines

To generate *Tg(-0.8myl7:H2B-mScarlet)*, the H2B and mScarlet sequences were cloned into a Tol2 backbone downstream of 800 bp of the *myl7* promoter (*-0.8myl7*). Cloning was performed using InFusion Cloning (Takara Bio). The construct was injected into AB embryos at the one-cell stage (30 pg/embryo) together with *Tol2* mRNA (25 pg/embryo) to establish the lines.

### Generation of mutant lines using CRISPR/Cas9

To generate the *tcf21^bns427^* and *wt1a^bns428^* mutant lines, the CRISPR design tool CHOPCHOP (https://chopchop.cbu.uib.no/) was used to design short guide RNAs (sgRNAs). The sgRNAs were assembled as described previously (Gagnon et al., 2014; Varshney et al., 2015) and transcribed using a MegaShortScript T7 Transcription Kit (Thermo Fisher Scientific). *cas9* mRNA was transcribed using an mMESSAGE mMACHINE T3 Transcription Kit (Thermo Fisher Scientific) using pT3TS-nCas9n (Addgene plasmid #46757) as a template. sgRNAs and *cas9* RNAs were purified with an RNA Clean and Concentrator Kit (Zymo Research). gRNAs (75 pg/embryo *tcf21* gRNA; 25 pg/embryo *wt1a* gRNA) and *cas9* mRNA (∼200 pg/embryo) were co-injected at the one-cell stage.

For *tcf21^bns427^*, an sgRNA targeting exon 1 (targeting sequence, 5′-CGCAGCTAACGCGCGC GAGA-3′) was used, resulting in a 10 bp deletion. To generate the *wt1a^bns428^* promoter-less allele, three sgRNAs targeting the promoter region of *wt1a* were designed and co-injected: two sgRNAs targeting the proximal promoter (sgRNA1, 5′-CAACTGGACAGCTTGGCCTA-3′; sgRNA2, 5′-AAAGGCGTCTAATAGACAAC-3′) and one sgRNA targeting intron 1 (sgRNA3, 5′-GGCAGTGCCACTCTTGCCAG-3′), resulting in a deletion of 8 kb.

*tcf21* mutants were genotyped using high resolution melt analysis (Eco Illumina) with the following primers: *tcf21* HRM fw, 5′-GCAAGCAGGTCCAGAGGA-3′, and *tcf21* HRM rv, 5′-ACGGTAACGTCGTCTTCAGC-3′. *tcf21* mutants carrying *TgBAC(tcf21:NLS-EGFP)* were additionally genotyped by PCR, to distinguish heterozygous and homozygous mutant embryos, using the following primers: *tcf21* PCR fw, 5′-TGTCTCCAGCCAACATGTCCA-3′, and *tcf21* PCR rev, 5′-GCGCATCCTCGCCCTCTCG-3′.

*wt1a* mutants were genotyped by PCR combining a common reverse primer (*wt1a* 3′ rv common, 5′-GCTGATCATCTCTGCGTTTG-3′) with one forward primer in the promoter region upstream of the deletion (*wt1a* 5′ fw1, 5′-TGTGAAATGAATGACACATCAAG-3′) or one forward primer in intron 1 inside the deleted region to detect the WT allele (*wt1a* 3′ fw2, 5′-CAATTGAAAAACTTTAAAAATCAGCA-3′).

### Chemical treatments

For cell ablation experiments, metronidazole (MTZ, Sigma-Aldrich) treatment was performed as described previously (Curado et al., 2007, 2008; Pisharath et al., 2007; Wang et al., 2015) with some modifications. MTZ powder was freshly dissolved in DMSO at 1 M concentration and subsequently diluted in egg water to 5 mM (52-80 hpf, 52-100 hpf) or 7 mM (96-144 hpf) concentration. Embryos and larvae were treated in different time windows (52-80 hpf, 52-100 hpf, 96-144 hpf). The larvae were then immediately imaged or rinsed with egg water before growing in the incubator.

The broad FGFR inhibitor (BGJ398, Selleck Chemicals) (De Simone et al., 2021) and the VEGFR inhibitor (SKLB1002, Selleck Chemicals) (Zhang et al., 2011) were dissolved in DMSO at a concentration of 10 and 5 mM, respectively, and frozen in single-use aliquots at −80°C. Larvae were treated with a final concentration of 7.5 μM BGJ398 or 2.5μM SKLB1002 (Matsuoka et al., 2016). Different concentrations were first tested to minimize off-target developmental defects and the lowest effective doses were chosen. Control embryos were treated with the same concentrations of DMSO.

### Whole-mount immunostaining

Whole-mount immunostaining was performed as described (Boezio et al., 2020). Larvae were fixed in 4% paraformaldehyde for 2 h at room temperature after stopping the heart with 0.4% tricaine. The fixative was substituted with PBS containing 0.1% Tween-20 and the yolks were manually removed using forceps. The blocking step preceding primary antibody incubation was performed in PBS with 1% bovine serum albumin, 1% DMSO and 0.5% Triton X-100 supplemented with 5% goat serum. Primary antibody incubations were performed overnight at 4°C at the following dilutions: anti-GFP (1:400, chicken; cat. no GFP-1020, AvesLabs), anti-Cav1 (1:50, mouse; cat. no 610406, BD Biosciences) and DsRed polyclonal antibody (1:200, rabbit; cat. no. 632496, Takara). All secondary antibodies tagged with Alexa Fluor 568, Alexa Fluor 488 and Alexa Fluor 647 (Thermo Fisher Scientific) were incubated overnight at 4°C at a 1:500 dilution. Larvae were incubated with 1 µg/ml DAPI with the secondary antibody.

### Whole-mount *in situ* hybridization and fluorescence *in situ* hybridization

The following primers were used to generate the DNA template for the *in situ* RNA probes: *tcf21* ISH fw, 5′-CGCATGACACGTTTCCACAT-3′; *tcf21* ISH T7 rv, 5′-GGTAATACGACTCACTATAGGTGACATGACACTCGGCGT-3′; *wt1a* ISH fw, 5′-AAATGGCGTCACAGTTGGAG-3′; *wt1a* ISH T7 rv, 5′-GGTAATACGACTCACTATAGGTGTAATCAATCGACCTGCAGTG-3′; *fgf24* ISH fw, 5′-ATGTCTGTTCTGCCGTCAAGG-3′; and *fgf24* ISH T7 rv, 5′-TAATACGACTCACTATAGGAGTTTGTATTGGGGTTGGGT-3′. Digoxigenin (DIG)-labeled probes were transcribed *in vitro* from PCR products, using T7 polymerase (Promega) and a DIG RNA labeling kit (Roche).

*In situ* hybridization (ISH) was performed as described previously (Thisse and Thisse, 2008). Anti-MF20 primary antibody [mouse, 1:100, Developmental Studies Hybridoma Bank (DSHB); MF 20 was deposited to the DSHB by D.A. Fischman (DSHB Hybridoma Product MF 20)] to label the heart was added together with the anti-DIG antibody (1:5000, cat. no. 11093274910, Roche). The secondary antibody goat anti-mouse Alexa Fluor 488 (1:500, cat. no. A-11001, Thermo Fisher Scientific) was added after treatment with BM Purple (Roche) for 2 h at room temperature and washed in PBS with 0.1% Tween-20 before imaging.

Fluorescence *in situ* hybridization (FISH) was performed as described previously (Gunawan et al., 2019) and following the same protocol as for ISH, with some modifications. Briefly, after the hybridization with the probe and the blocking step, larvae were incubated with anti-DIG-POD (1:500, cat. no. 11207733910, Roche), anti-dsRed (rabbit, 1:200; cat. no. 632496, Takara), and anti-MF20 (mouse, 1:500; cat. no. 14-6503-82, Invitrogen) primary antibodies at 4°C overnight. After several washes with PBS containing 0.1% Tween-20, larvae were incubated in amplification buffer with TSA fluorescein reagent (1:100; Roche) at 37°C for 1-2 h. Samples were protected from light during the staining reaction. The reaction was stopped by a wash with PBS with 0.1% Tween-20 (15 min) followed by a 6% H_2_O_2_ wash for 30 min.

Larvae were incubated with goat anti-rabbit Alexa Fluor 647 and goat-anti mouse Alexa Fluor 568 (1:500, cat. no. A-21244, A-11004, Thermo Fisher Scientific) and DAPI (10 µg/ml) for 2 h at room temperature and washed in PBS with 0.1% Tween-20 before imaging with a Nikon SMZ25 stereomicroscope with a 2×/0.3 objective (ISH) or a Zeiss LSM880 confocal microscope with a 20× objective (FISH).

### Confocal microscopy imaging

After immunostaining, fixed larvae were mounted in 1% low-melting agarose/egg water without tricaine, imaged with a Zeiss LSM700 confocal microscope with a W Plan-Apochromat 40×/1.0 dipping lens, and genotyped after imaging. For confocal imaging of live animals, larvae were embedded in 1% low-melting agarose with 0.2% or 0.01% tricaine to image stopped or beating hearts, respectively.

Videos of beating hearts were acquired with an exposure time of 5 ms for 20-40 s with a Zeiss spinning-disk confocal microscope. No tricaine was added to the agarose to avoid alteration of the heart rate. Confocal imaging of live hearts was performed with a Zeiss LSM700 or LSM800 Observer confocal microscope. Ventricular fractional shortening was quantified with ImageJ, using the following formula: [(width at diastole−width at systole)/width at diastole]×100.

### Mosaic *myl7:mScarlet* analyses

The *myl7:mScarlet* plasmid (Uribe et al., 2018) was injected at the one-cell stage (25 pg/embryo) together with *Tol2* mRNA (25 pg/embryo) into *Tg(myl7:BFP-CAAX)* embryos derived from *tcf21^+/−^* intercrosses. Injected embryos were selected for fluorescence at approximately 48 hpf. The same larvae were consecutively imaged at 76 and 100 hpf. Considering the variability in size and shape of the CMs depending on their location (Auman et al., 2007; Priya et al., 2020), only *myl7:mScarlet^+^* compact-layer CMs in the ventricular outer curvature were analyzed. The larvae were genotyped after imaging and analysis.

### Image analyses

The CM apical areas were manually quantified on a 3D reconstruction of the entire heart in ImageJ. CMs with inactive ERK signaling were defined as CMs with nuclear enrichment of the Clover signal and manually quantified in 2D sections in ImageJ. The numbers of total CMs, trabecular CMs, Venus-Gmnn^+^ CMs and EpiCs, and average internuclear distance were analyzed using the Spots function of the Imaris (Bitplane) software. 3D cardiac surface rendering, ventricular volume and individual CM volume quantifications were obtained with the Surfaces function of the Imaris (Bitplane) software. The numbers of extruding CM were quantified on the 3D myocardial surface rendering. Illustrations were done in Inkscape (XQuartz X11).

### Statistical analyses

All statistical analyses were performed in GraphPad Prism (Version 8.4). Samples were tested for normal distribution using the D'Agostino–Pearson normality test. The following parametric tests were performed: unpaired two-tailed Student's *t*-test or one-way ANOVA, followed by Dunnett’s multiple comparison test, unless specified otherwise, for comparison of 2 or ≥3 samples, respectively. Non-parametric tests used were the Mann–Whitney test or Kruskal–Wallis test, followed by Dunn's multiple comparison test, for the comparison of 2 or ≥3 samples, respectively.

### Randomization and blinding procedures

After selecting embryos or larvae for the relevant fluorescence signal, they were allocated randomly to different experimental groups. Animals were collected, grown or processed in the same dish or tube and genotyped after the imaging and analysis. For all experiments using the NTR-MTZ system, embryos and larvae from outcrosses of *TgBAC(tcf21:mCherry-NTR); Tg(myl7:EGFP-HRAS)* and AB were used. For the experiments performed to assess the epicardial coverage of MTZ-treated fish (Fig. 1M,N, Fig. S1, Fig. 3), DMSO-treated larvae were used as control. For most other experiments, all larvae were treated with MTZ to exclude contribution of the MTZ treatment to the observed phenotypes. *TgBAC(tcf21:mCherry-NTR)^−^* (*tcf21:*NTR^−^) larvae were used as controls and compared with *TgBAC(tcf21:mCherry-NTR)^+^* (*tcf21:*NTR^+^) larvae. For the experiments shown in Fig. 1L and Fig. 3G-I designed to count surviving EpiCs after ablation, both *tcf21:*NTR+ DMSO-treated larvae and *tcf21:*NTR^−^ MTZ-treated larvae were used as controls, and no significant difference was observed between the two groups. Therefore, the samples were pooled into the control group. Whenever possible, the investigators were blinded to allocation during experiments, data collection and analyses.

### Heart isolation and fluorescence-activated cell sorting

Hearts from 96 hpf *TgBAC(tcf21:NLS-EGFP); Tg(myl7:mCherry-CAAX)* larvae were manually dissected in Dulbecco's modified Eagle's medium (DMEM, cat. no. 21969-035, Gibco) with 10% fetal bovine serum (FBS, S0615, Sigma/Merck). *tcf21^−/−^* larvae, derived from *tcf21^+/−^* intercrosses, were selected based on the phenotype (i.e. lack of head muscles), whereas *tcf21^+/+^* larvae were obtained from *tcf21^+/+^* sibling intercrosses. To minimize bias in our analyses and to collect enough ventricular EpiCs to investigate the EpiC-CM crosstalk, we selected only *tcf21^−/−^* larvae that exhibited a substantial ventricular epicardial coverage. Although this choice might have led to the selection of larvae with milder transcriptomic differences, we observed that these hearts displayed a morphologically comparable phenotype with the *tcf21* mutants with less ventricular epicardial coverage (Fig. S4D,F). Hearts were centrifuged for 60 s at 2300 ***g*** and collected, washed with 1 ml Hanks' Balanced Salt Solution (HBSS) and dissociated into single cells by incubating in 100 μl Enzyme 1 and 5 μl Enzyme 2 (Pierce Cardiomyocytes Dissociation Kit, Thermo Fisher Scientific) for 20 min at 300-350 rpm in a 30°C shaker. The digestion was stopped by adding 1 ml DMEM with FBS. After centrifuging for 3 min at 800 ***g*** and discarding the supernatant, fresh DMEM with FBS was added to the dissociated cells and passed through 40 μl-filtered fluorescence-activated cell sorting (FACS) tubes. Cell viability was assessed with DAPI. Negative controls consisting of non-fluorescent hearts or single-color fluorescent hearts were prepared to adjust the gating. Cell filtering was performed by forward scatter amplitude (FSC-A) versus side scatter amplitude (SSC-A). Notably, from the FACS analyses, we observed that a population of *tcf21^+^* cells was also mCherry^+^ (Fig. S5A). We confirmed, through confocal imaging (Fig. S5B,B′), that a subset of ventricular *tcf21^+^* EpiCs displayed low levels of *Tg(myl7:*mCherry-CAAX*)* expression. According to this confocal imaging analysis, the double-positive population was composed of ventricular EpiCs. The single *TgBAC(tcf21:NLS-EGFP)^+^* population was likely composed of OFT EpiCs and some ventricular EpiCs not expressing the *myl7* transgene. In order to sort a sufficient number of ventricular EpiCs necessary to investigate the ventricular intercellular crosstalk between EpiCs and CMs, we decided to include the double-positive cells in the sorted epicardial population.

*TgBAC(tcf21:NLS-EGFP)^+^* cells (EpiCs) and *Tg(myl7:EGFP-HRAS)^+^/ TgBAC(tcf21:NLS-EGFP)^−^* cells (CMs) were sorted on a FACSAria III machine (BD Biosciences), directly collected in TRIzol, and frozen at −80°C before RNA sequencing. Approximately 7000-10,000 EpiCs and CMs were collected per biological replicate. The same number of cells was collected per genotype, over five sorting sessions.

### Transcriptomic analysis

Total RNA from sorted cells was isolated using the miRNeasy micro kit (QIAGEN), combined with on-column DNase digestion (DNase-Free DNase Set, QIAGEN). Total RNA and library integrity were verified with LabChip Gx Touch 24 (Perkin Elmer). Approximately 4 ng of total RNA was used as input for SMART-Seq v4 Ultra Low Input RNA Kit (Takara Clontech) for cDNA pre-amplification. Obtained full-length cDNA was checked on LabChip and fragmented by Ultrasonication using an E220 machine (Covaris). Final library preparation was performed using a Low Input Library Prep Kit v2 (Takara Clontech). Sequencing was performed on a NextSeq500 instrument (Illumina) using v2 chemistry, resulting in an average of 37 million reads per library with 1×75 bp single end setup. The resulting raw reads were assessed for quality, adapter content and duplication rates with FastQC (http://www.bioinformatics.babraham.ac.uk/projects/fastqc). Trimmomatic version 0.39 was used to trim reads with a quality drop below a mean of Q20 in a window of ten nucleotides (Davis et al., 2013). Only reads between 30 and 150 nucleotides were used in subsequent analyses. Trimmed and filtered reads were aligned versus the Ensembl Zebrafish genome version DanRer11 (ensemble release 99) using STAR 2.7.3a with the parameter ‘outFilterMismatchNoverLmax 0.1’ to set the maximum ratio of mismatches to mapped length at 10% (Dobin et al., 2013). The number of reads aligning to genes was counted with featureCounts 1.6.5 tool from the Subread package (Liao et al., 2014). Only reads mapping at least partially inside exons were admitted and aggregated per gene, whereas reads overlapping multiple genes or aligning to multiple regions were excluded from further analyses. Differentially expressed genes were identified using DESeq2 version 1.26.0 (Love et al., 2014). Genes were classified to be differentially expressed genes (DEGs) with the Benjamini–Hochberg-corrected *P*-value<0.05 and −0.59≤ Log_2_FC≥+0.59. The Ensembl annotation was enriched with UniProt data (release 06.06.2014) based on Ensembl gene identifiers. The top DEGs among the different samples are listed in Tables S2 and S3.

Differentially expressed genes encoding secreted factors were manually curated and annotated based on the Zebrafish matrisome genes (Nauroy et al., 2018). Expression data were imported in Python, Z-score transformed and plotted as heatmaps using Seaborn (https://joss.theoj.org/papers/10.21105/joss.03021).

### Gene Ontology analyses

GO enrichment of differentially expressed genes (log_2_FC>|0.585|; *P*<0.05) of individual sub-ontologies (Biological Processes, Molecular Functions and Cellular Components) was calculated using Cluster Profiler (v 4.4.0) (Yu et al., 2012) and with annotations derived from org.Dr.eg.db (v 3.14.0). For all Gene Set Enrichment analyses, the R package fgsea (v.1.20.0) was used.

Scripts and data are available at https://github.com/giulia-boezio/epicardium_analyses. The data used for the plots, including individual gene names for each GO term, are available in Table S4.

### RT-qPCR

Expression of *tcf21* and *wt1a* was analyzed in single 96 hpf larvae deriving from *tcf21^bns427^* or *wt1a^bns428^* heterozygous intercrosses, respectively. DNA and RNA were extracted from single embryos using TRIzol reagent, followed by TRIzol-chloroform extraction, as described previously (El-Brolosy et al., 2019).

To analyze *fgf24* and *vegfaa* expression, hearts were manually dissected from 96 hpf *tcf21^+/+^* and *tcf21^−/−^* larvae. *tcf21^−/−^* larvae were selected based on the lack of head muscles. RNA was extracted with the standard TRIzol-chloroform extraction and purified with RNA Clean and Concentrator Kit (Zymo Research).

Approximately 500 ng mRNA was used to synthesize cDNA using the Maxima First Strand cDNA kit (Thermo Fisher Scientific). For all experiments, DyNAmo ColorFlash SYBR Green qPCR Mix (Thermo Fisher Scientific) was used on a CFX connect Real-time System (Bio-Rad). All reactions were performed in technical triplicates and from three or more biological replicates. Every biological replicate consisted of cDNA from five to ten larvae or from 10-12 hearts per genotype. Gene expression values were normalized to the the zebrafish *rpl13a* housekeeping gene. The primers used are listed in Table S1.

## Supplementary Material

10.1242/dmm.049571_sup1 Supplementary information
