# Supplementary material for: The developing epicardium regulates cardiac chamber morphogenesis by promoting cardiomyocyte growth
Source: Dis Model Mech. 2022 Oct 19;16(5):dmm049571. doi: 10.1242/dmm.049571 (PMC9612869; doi:10.1242/dmm.049571)
Supplement: Supplementary information [file dmm-16-049571-s1.pdf]

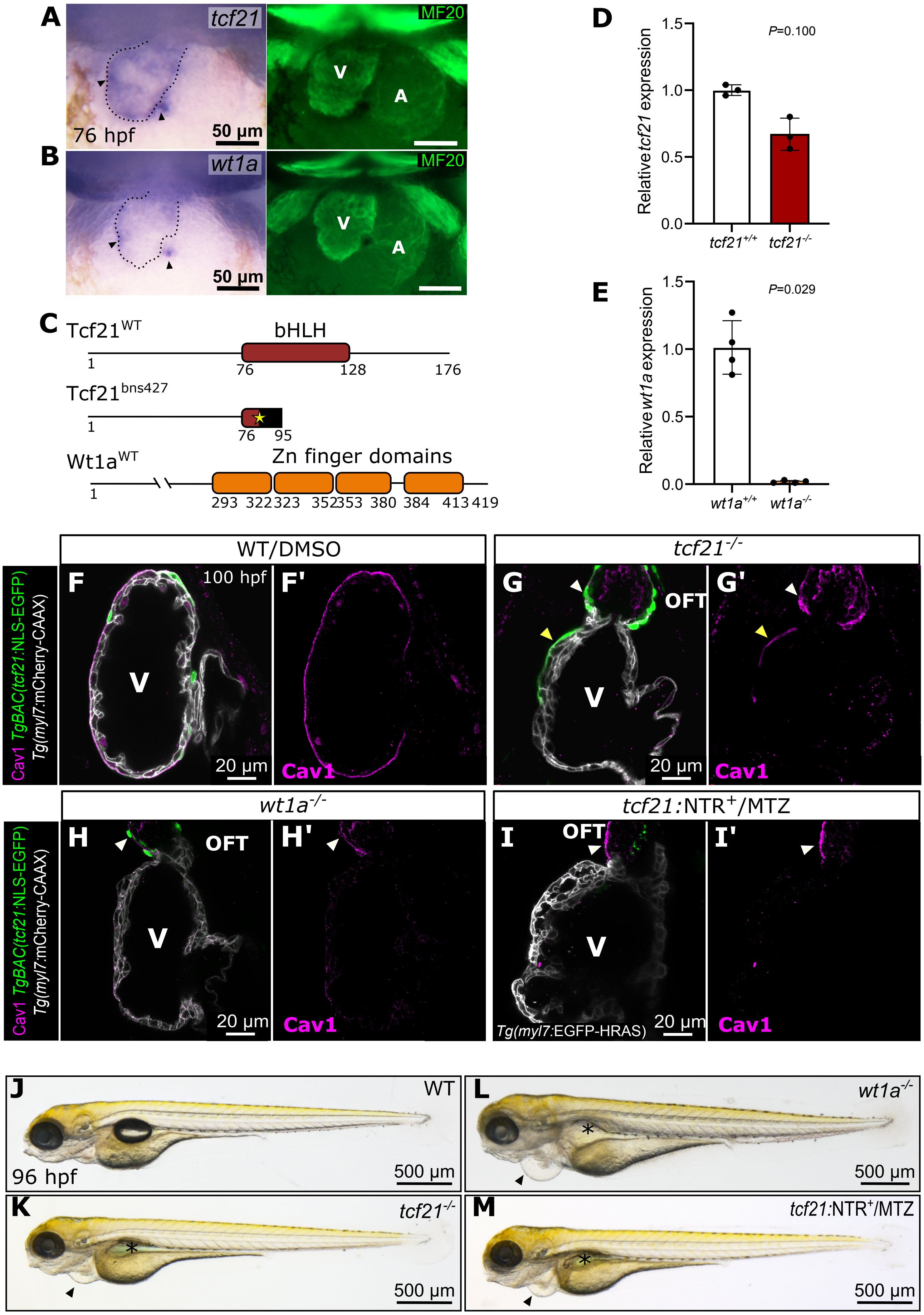
A
tcf21
76 hpf
50 µm
MF20
V
A
B
wt1a
50 µm
MF20
V
A
C
Tcf21WT
bHLH
1
76
128
176
Tcf21bns427
1
76
95
Wt1aWT
Zn finger domains
1
293 322 323 352 353 380 384 413 419
D
P=0.100
Relative tcf21 expression
1.5
1.0
0.5
0.0
tcf21+/+
tcf21-/-
E
P=0.029
Relative wt1a expression
1.5
1.0
0.5
0.0
wt1a+/+
wt1a-/-
Cav1 TgBAC(tcf21:NLS-EGFP) Tg(myl7:mCherry-CAAX)
WT/DMSO
F
100 hpf
V
20 µm
F'
Cav1
tcf21-/-
G
OFT
V
20 µm
G'
Cav1
wt1a-/-
H
OFT
V
20 µm
H'
Cav1
tcf21:NTR+/MTZ
I
OFT
V
Tg(myl7:EGFP-HRAS)
20 µm
I'
Cav1
J
WT
96 hpf
500 µm
L
wt1a-/-
500 µm
K
tcf21-/-
500 µm
M
tcf21:NTR+/MTZ
500 µm

Fig. S1. Transcription factors Tcf21 and Wt1a are crucial for cardiac development A, **B**) *In situ* hybridization showing the expression of *tcf21*(A) and *wt1a* (B) in 76 hpf hearts. MF20 immunostaining (green) labels the myocardium (dashed lines); arrowheads point to the presumptive signal in the epicardium. **C**) Schematics of wild-type Tcf21 and Wt1a proteins and predicted Tcf21 mutant protein, highlighting the bHLH (red/brown, Tcf21) and Zn finger (orange, Wt1a) domains. Yellow star indicates the CRISPR/Cas9-induced mutation site; the black rectangle represents new sequence downstream of the frameshift-inducing mutation. The *wt1a* mutation targets the promoter region and does not affect the coding sequence. **D,** E) *tcf21* (D) and *wt1a* (E) mRNA levels in 96 hpf *tcf21*$^{+/+}$ and *tcf21*$^{-/-}$ larvae (D) and in *wt1a*$^{+/+}$ and *wt1a*$^{-/-}$ larvae (E); means ± SD; P values from Mann Whitney test; Ct values are listed in Table S1. **F-I'**) Confocal images of 100 hpf hearts immunostained for Caveolin1 (Cav1). Cav1 immunostaining is present only in “escaper” ventricular *tcf21*$^{+}$ EpiCs (yellow arrowheads, *tcf21*$^{-/-}$) and in the OFT (white arrowheads). **J-M**) 96 hpf *tcf21*$^{-/-}$, *wt1a*$^{-/-}$, and *tcf21:*NTR^{+} MTZ-treated larvae exhibit pericardial edema (arrowheads) and lack of swim bladder inflation (asterisks). A, atrium; V, ventricle; OFT, outflow tract; bHLH, basic helix–loop–helix; Zn, zinc.

Disease Models & Mechanisms • Supplementary information

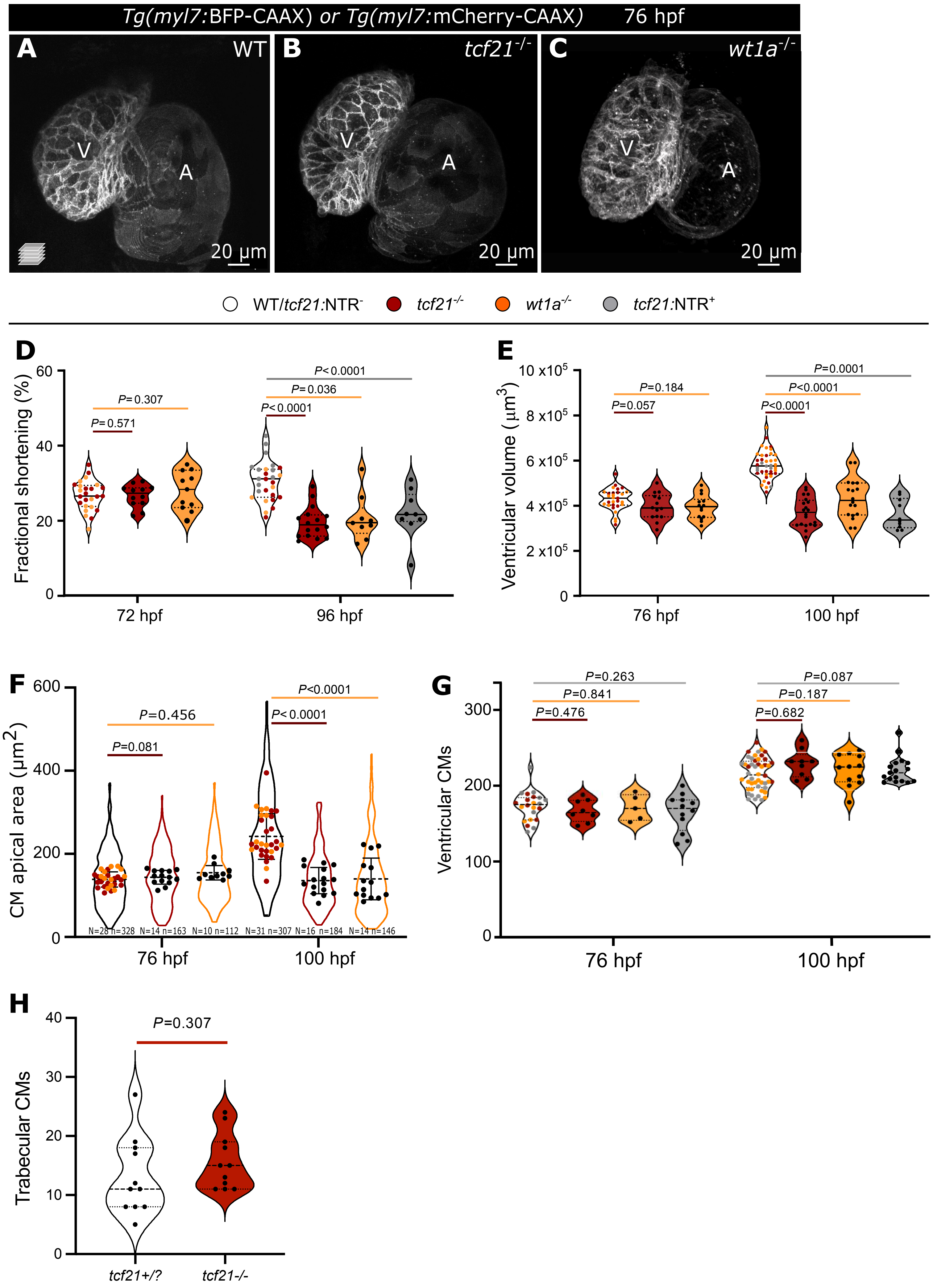

Disease Models & Mechanisms • Supplementary information

Fig. S2. Epicardial impairment affects ventricular size, but not ventricular cardiomyocyte number

A-C) Confocal images of 76 hpf WT, *tcf21*$^{-/-}$, and *wt1a*$^{-/-}$ larvae, all exhibiting a similar ventricular size. **D-E**) Quantification of fractional shortening (D) and ventricular volume (E) in WT, mutant and *tcf21:*NTR^{+} MTZ-treated larvae; the graph in E relates to Figure 2E, showing the single data points. **F**) CM apical area in WT, *tcf21*$^{-/-}$, and *wt1a*$^{-/-}$ larvae; related to Figure 2F. Violin plot represents the distribution of individual CMs (n); dots represent the average per larva (N). **G**) Ventricular CM numbers in WT, mutant and *tcf21:*NTR^{+} MTZ-treated larvae, showing individual data points; related to Figure 2J. **H**) Trabecular CMs in 96 hpf *tcf21*$^{+/+}$ and *tcf21*$^{-/-}$ larvae, showing individual data points. **A, B, F, G**) The colors of wild-type dots refer to *tcf21*$^{+/+}$ (brown), *wt1a*$^{+/+}$ (orange), and *tcf21:*NTR^{-} MTZ-treated (grey) siblings. Median and quartiles (A, B, G), or mean ± SD (F); *P* values from *t*- or Mann-Whitney test (following normality test), compared with the WT/ control siblings of each genotype/treatment. WT, wild type; A, atrium; V, ventricle; N, number of larvae; n, number of CMs.

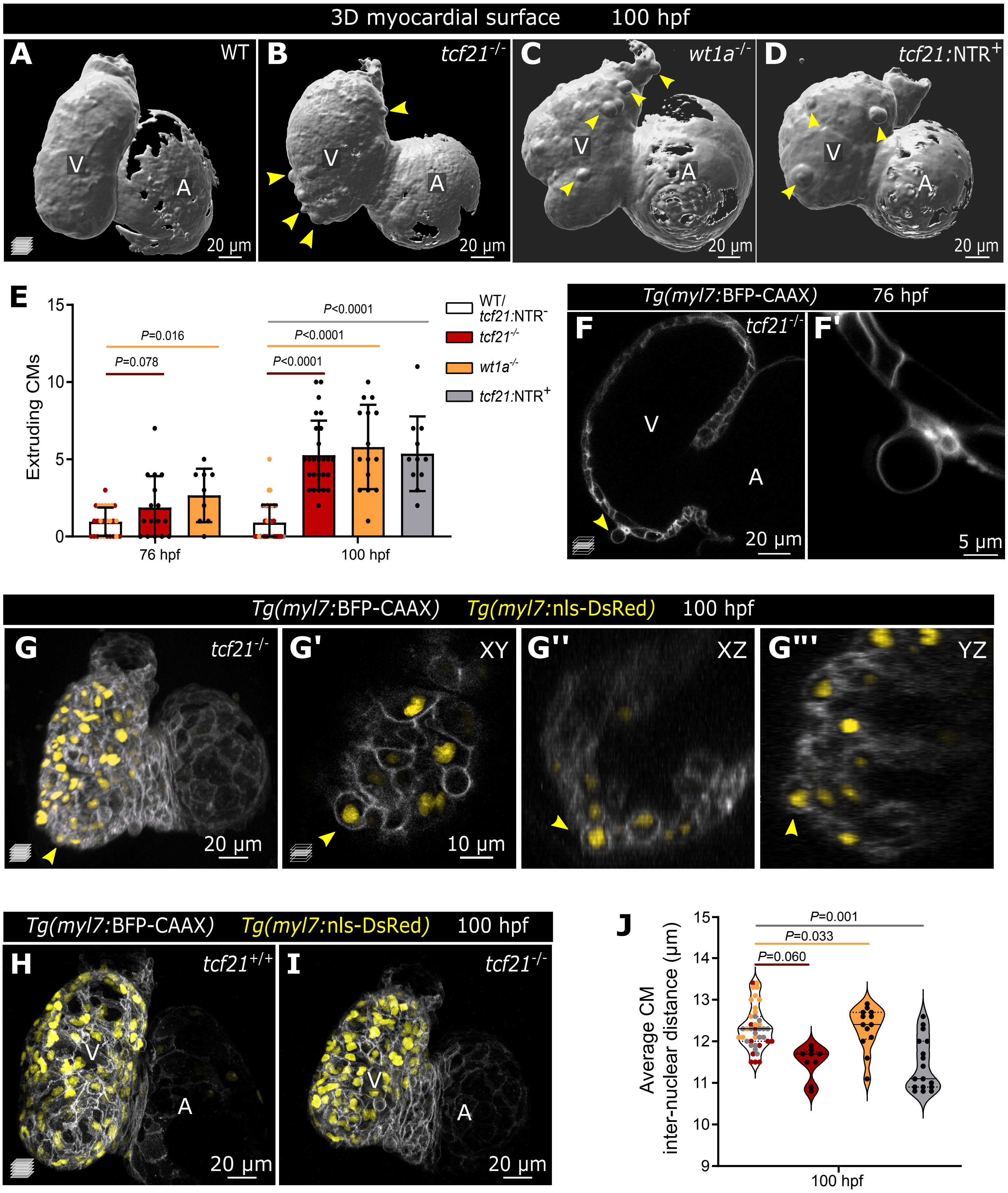

Disease Models & Mechanisms • Supplementary information

Fig. S3. Impaired epicardial coverage causes abluminal cardiomyocyte extrusion A-D) 3D surface rendering of 96 hpf *Tg(myl7:mCherry-CAAX)* (A-C) and *Tg(myl7:EGFP-HRAS)* (D) hearts. Arrowheads point to extruding CMs. **E**) Quantification of CM extrusions at 76 and 96 hpf. **F, F'**) Single-plane images of 76 hpf *Tg(myl7:mCherry-CAAX); tcf21$^{-/-}$* heart. Arrowhead points to extruding CM. **G-G'''**) Confocal images showing an intact nucleus in an extruding CM (yellow arrowheads) in a 100 hpf *Tg(myl7:BFP-CAAX); (myl7:nlsDsRed)* heart. G'-G''' are magnified images of G as seen from different orientations. **H-J**) Confocal images and quantification of the CM inter-nuclear distance in 100 hpf *Tg(myl7:BFP-CAAX); (myl7:nlsDsRed)* hearts. **E, J**) The colors of wild-type dots refer to *tcf21*$^{+/+}$ (brown) or *wt1a*$^{+/+}$ (orange), and *tcf21:*NTR^{-} MTZ-treated (grey) siblings. Mean ± SD (E) or median and quartiles (J); *P* values from *t*- or Mann-Whitney test (following normality test), compared with WT/control siblings of each genotype/treatment. A, atrium; V, ventricle.

Disease Models & Mechanisms • Supplementary information

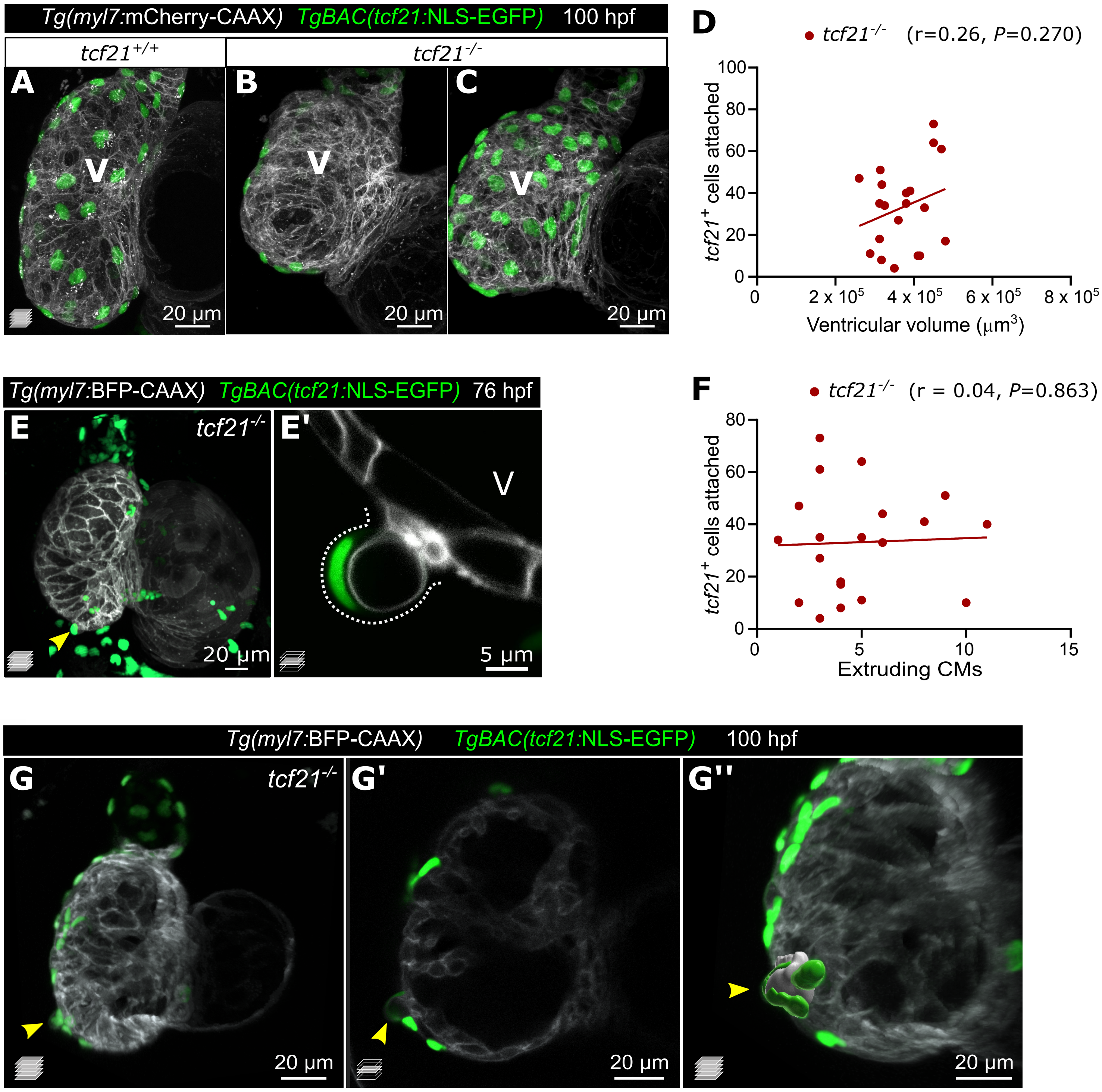

Disease Models & Mechanisms • Supplementary information

Fig. S4. An intact epicardium is required to promote ventricular growth and prevent cardiomyocyte extrusion

A-C) Confocal images of 96 hpf *Tg(myl7:BFP-CAAX); TgBAC(tcf21:NLS-EGFP); tcf21*$^{+/+}$ (A) and *tcf21*$^{-/-}$ (B, C) hearts, with different degrees of epicardial coverage (green). **D, F**) Pearson correlation between ventricular volume (D) and the number of extruding CMs (F) (X axis), and the number of ventricular *tcf21*$^{+}$ cells (Y axis) in 96 *tcf21*$^{-/-}$ larvae. **E, E'**) Single confocal plane of a 76 hpf *TgBAC(tcf21:NLS-EGFP); Tg(myl7:mCherry-CAAX) tcf21*$^{-/-}$ ventricle exhibiting an extruding CM covered by a *tcf21*$^{+}$ epicardial cell (nucleus, green; cell body highlighted with dashed line). The same larva is shown in Fig. S3F. **G-G''**) Confocal images of a 100 hpf *Tg(myl7:mCherry-CAAX); TgBAC(tcf21:NLS-EGFP); tcf21*$^{-/-}$ ventricle exhibiting an extruding CM covered by a *tcf21*$^{+}$ epicardial cell (yellow arrowheads). G'' shows a 3D surface rendering of the CM and EpiCs highlighted by the arrowhead. V, ventricle.

Disease Models & Mechanisms • Supplementary information

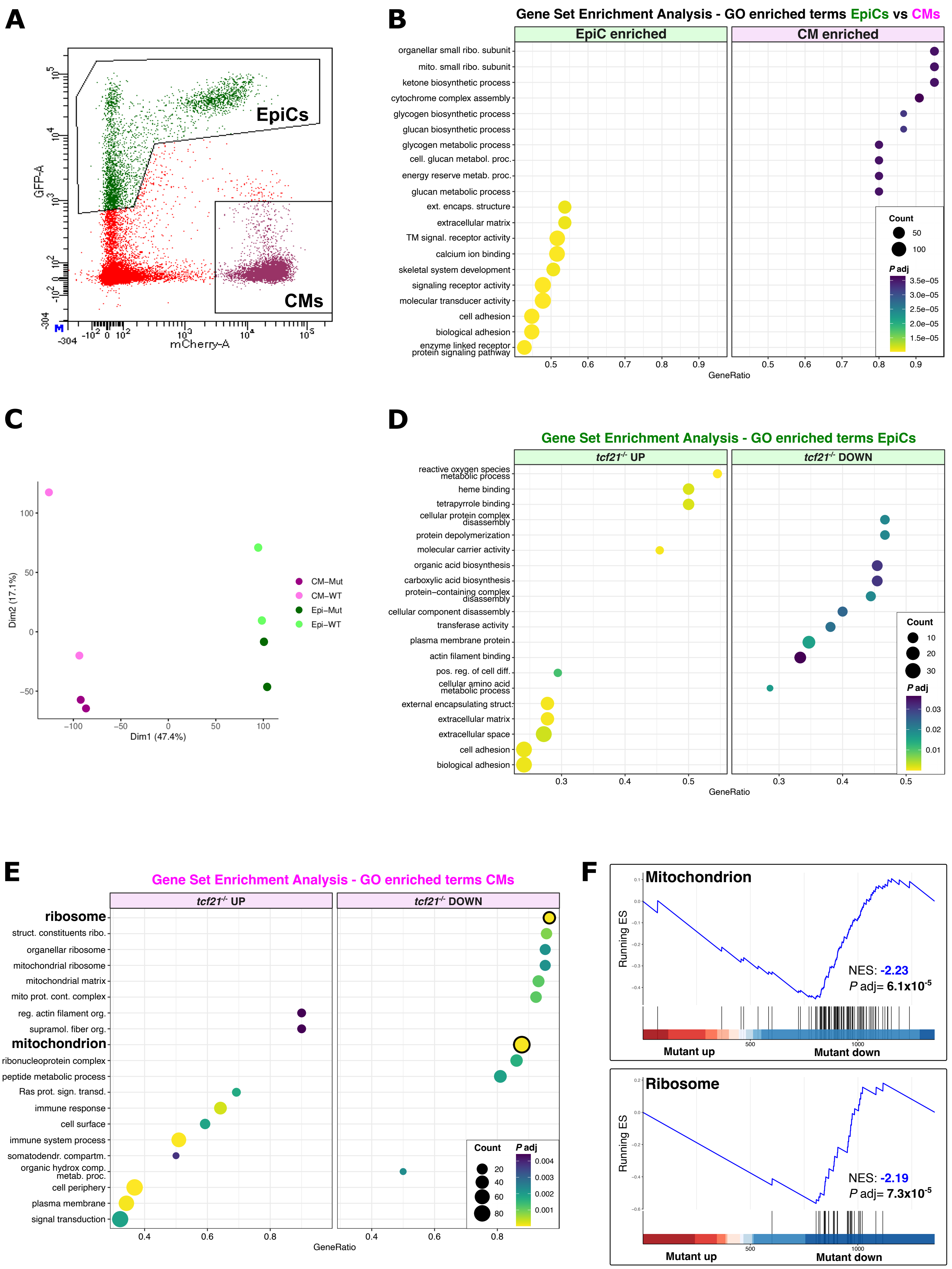

Disease Models & Mechanisms • Supplementary information

Fig. S5. Transcriptomic analysis of sorted t*cf21*$^{+/+}$ and *tcf21*$^{-/-}$ EpiCs and CMs

A) Fluorescence-activated cell sorting graph depicting the gating selected to sort EpiCs (green; GFP+) and CMs (purple; mCherry+, GFP-). See Materials and Methods for details on the sorted populations. **B, D, E**) Gene Set enrichment analysis (GSEA) bubble plot, showing the most overrepresented Gene Ontology (GO) terms enriched in *tcf21*$^{+/+}$ EpiCs or CMs (B), up- or downregulated in *tcf21*$^{-/-}$ EpiCs (**D**) and CMs (**E**). **C**) Dimensionality reduction (Principal Component Analysis, PCA) of the RNA-seq experiment represented in Fig. 4A. Each point represents an RNA-seq sample. **F**) GSEA plots for the two most overrepresented Gene Ontology terms amongst the downregulated genes in *tcf21*$^{-/-}$ CMs, showing also their normalized enrichment score (NES) and *P* adjusted value. GO, Gene ontology; GSEA, gene set enrichment analysis; PCA, principal component analysis.

Disease Models & Mechanisms • Supplementary information

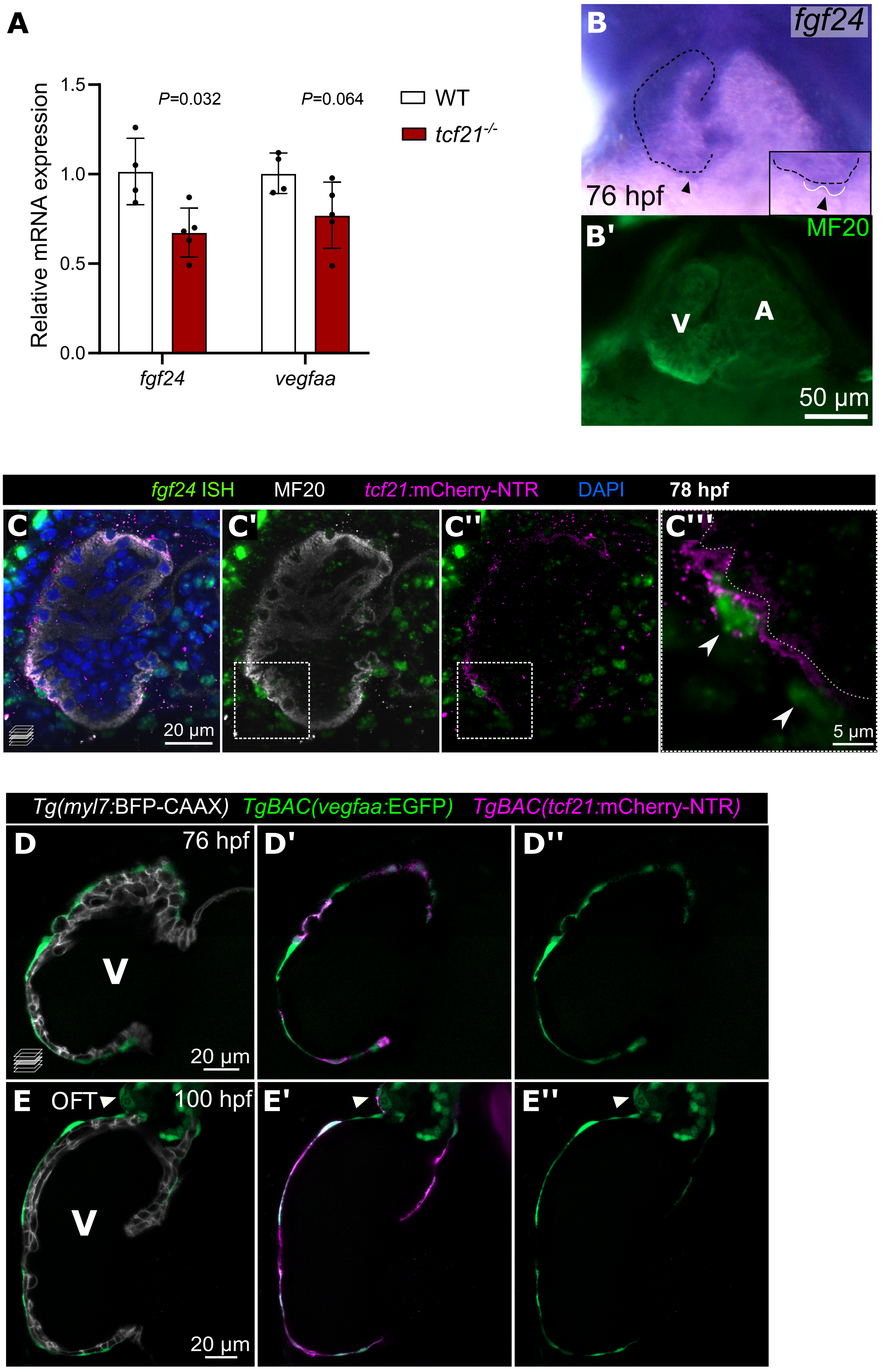

Disease Models & Mechanisms • Supplementary information

Fig. S6. *fgf24* and *vegfaa* expression is enriched in epicardial cells and downregulated in *tcf21*$^{-/-}$ hearts

A) *fgf24* and *vegfaa* mRNA levels obtained by RT-qPCR on extracted 96 hpf *tcf21*$^{+/+}$ and *tcf21*$^{-/-}$ hearts; means ± SD; *P* values from Mann Whitney test; Ct values are listed in Table S1. **B, B'**) *In situ* hybridization showing *fgf24* expression in 76 hpf hearts (n=11/11). MF20 immunostaining (green) labels the myocardium (dashed lines). Arrowheads, epicardial cells outside of the myocardial wall (enlarged in the box). **C-C''')** Fluorescence *in situ* hybridization showing *fgf24* expression in 78 hpf hearts (n = 8/8) and its enrichment in the epicardium (C''', arrowheads). MF20 immunostaining (white) labels the myocardium and *tcf21:*mCherry-NTR expression labels the epicardium. **D-E''**) Confocal images of 76 (D) and 100 (E) hpf *Tg(myl7:BFP-CAAX); TgBAC(tcf21:mCherry-NTR); TgBAC(vegfaa:EGFP)* ventricles. *TgBAC(vegfaa:*EGFP*)* expression is restricted to the epicardium (here seen on the ventricle and OFT) as well as OFT smooth muscle cells (white arrowheads). A, atrium; V, ventricle; OFT, outflow tract.

Table S1. Ct values of gene expression by RT-qPCR, and primer sequences.

Click here to download Table S1

Table S2. List of the top differentially expressed genes (>1 or <-1 log2FC, baseMean > 50) from RNA-seq datasets of 96 hpf CMs sorted from *tcf21*$^{+/+}$ and *tcf21*$^{-/-}$ larval hearts.

Click here to download Table S2

Table S3. List of the top differentially expressed genes (>1 or <-1 log2FC, baseMean > 50) from RNA-seq datasets of 96 hpf EpiCs sorted from *tcf21*$^{+/+}$ and *tcf21*$^{-/-}$ larval hearts.

Click here to download Table S3

Table S4. List of all Gene Ontology terms (shown in Figure S5, and related gene IDs) enriched in the different sorted populations, obtained from the RNA-seq datasets of 96 hpf *tcf21*$^{+/+}$ and *tcf21*$^{-/-}$ larval hearts.

Click here to download Table S4
